# Network‐based prediction and real‐world patient data observation identifies Doxycycline and Irbesartan as candidate medicines for Alzheimer’s disease and Traumatic Brain Injury

**DOI:** 10.1002/alz.089712

**Published:** 2025-01-09

**Authors:** Marina Bykova, Michael Danziger, Michal Rosen‐Zvi, Andrew A. Pieper, Feixiong Cheng

**Affiliations:** ^1^ Lerner Research Institute, Cleveland Clinic Foundation, Cleveland, OH USA; ^2^ Biological, Geological, and Environmental Sciences, Cleveland State University, Cleveland, OH USA; ^3^ IBM Research, Haifa Israel; ^4^ Case Western Reserve University, Cleveland, OH USA; ^5^ Cleveland Clinic, Cleveland, OH USA

## Abstract

**Background:**

Traumatic Brain Injury (TBI) is one of the most common nonheritable causes of Alzheimer’s disease (AD). However, there is lack of effective treatment for both AD and TBI. We posit that network‐based integration of multi‐omics and endophenotype disease module coupled with large real‐world patient data analysis of electronic health records (EHR) can help identify repurposable drug candidates for the treatment of TBI and AD.

**Method:**

We have collected a total of 69 TBI‐related genes and 144 AD‐related genes derived from human genetic and multi‐omics findings. We conducted network proximity analysis to prioritize likely drug candidates under the human protein‐protein interactome model. Using the MarketScan patient database (∼170 million individuals), the drugs were further investigated for association with a reduced incidence of AD against two patient cohorts: A cohort comprised of all patients over 70, and a TBI cohort comprised of patients who have sustained a traumatic brain, head, or facial injury.

**Result:**

Using network proximity‐based analysis of drug‐target networks and disease‐associated genes under the human protein‐protein interactome network, we identified 66 and 53 candidate drugs for AD and TBI, respectively (z‐score ≤ ‐3, p‐value ≤ 0.01). Drugs present within or related to drugs within both candidate lists, such as irbesartan and doxycycline, were especially prioritized for further real‐world patient data validation. In the subsequent Marketscan analysis, we found that doxycycline is significantly associated with a reduced incidence of AD in the over‐70 cohort as compared to cohort patients taking other anti‐infective and antibacterial drugs (OR = 0.92, adjusted 95% CI 0.88‐0.97, p‐value = 0.002). In addition, prescription of irbesartan was significantly associated with a reduced incidence of AD as compared to all cohort patients taking cardiovascular (OR = 0.82, adjusted 95% CI 0.71‐0.95, p‐value = 0.002) and renin‐angiotensin agents (OR = 0.82, adjusted 95% CI 0.71‐0.97, p‐value = 0.002).

**Conclusion:**

We demonstrated that combining network‐based predictions and large real‐world patient data observations has identified doxycycline and irbesartan as candidate treatments for AD. Further functional and clinical validation is warranted to examine a causal relationship between doxycycline and irbesartan with a future risk of developing AD.

